# Shared decision making in mental health: a novel approach

**DOI:** 10.36105/mye.2021v32n4.05

**Published:** 2021-10

**Authors:** Paola Buedo, Florencia Luna

**Affiliations:** * flasco Argentina. Buenos Aires, Argentina; ** flasco Argentina. Buenos Aires, Argentina

**Keywords:** Mental health, joint decision making, relational autonomy, health vulnerability, social stigma

## Abstract

The health system tends to underestimate the ability to make decisions of people with mental illnesses, characterizing them as vulnerable and adopting a stigmatizing attitude towards this vulnerability. Therefore, their autonomy, in the classical sense of the term, is reduced or nullified.

Another way to respond to vulnerability is by promoting autonomy, conceiving it as contextual and autonomy in a relational way. This could be beneficial for people with mental suffering because it allows analyzing what conditions could improve or harm the exercise of autonomy and consider the help of others in decision-making.

The shared decision-making process is a form of collaboration between professionals, patients and/or family members, in which the available evidence is shared with the patient and contextualized when faced with the task of making decisions in the medical environment.

## 1. Introduction

The decision-making capacity of people suffering from mental suffering tends to be underestimated (1, 2, and 3). Although in specific cases it is true that their capacity to make decisions is diminished by their condition, this is not the case for the majority of patients. However, on the latter assumption rests the prejudice about what a person with mental suffering can or cannot do (4).

This happens because, beyond the concrete capacities or potentialities that a person may have in the context of mental illness, there is a very close association between mental illness and social stigma (5, 6, 7, 8, 9), which is made up of different negative images, such as dangerousness, uselessness, impulsiveness, disability, among others, that undermine the patient’s decision-making capacity, moral agency and the exercise of his or her autonomy.

This implies a conceptual position situated in personal autonomy, from which it is stated that these persons are not autonomous, insofar as their cognitive capacity is restricted, and from vulnerability in its classic sense, indicating that all persons with mental disorders are vulnerable. These types of concepts eventually generate certain difficulties in the field of mental health, given that they sustain stigmatization and hinder or annul the decision making of people with mental illness.

This situation implies two a priori ethical problems: a) health professionals have a positive obligation in bioethical terms to promote autonomy, and one of its dimensions has to do with decision making, and b) the decisions made in the field of mental health regarding diagnosis and treatment have full repercussions on people’s way of life, since mental health as such allows us to develop our life, in the most holistic and complex form of the word (1, 10, 11).

When we go through a mental illness, life, the way of looking at other people, at projects, at the present and the future, even one’s own look, among others, will be modified by the suffering, by the way of naming it and by the way of approaching such suffering. This makes it essential to consider the person and his or her entire context, as well as his or her needs, desires and living conditions in mental health decision-making (13).

It is in this sense that we consider other analyses of the concepts of autonomy and vulnerability, such as relational autonomy and vulnerability thought from the notion of layers, as they are particularly useful in addressing these problems without reductionism or stigmatization. The first concept argues that social relations are a condition of possibility for autonomy, while the second explains that vulnerability is dynamic, closely related to the context and the specific relations of a given moment for a given person.

One of the approaches that would allow working both concepts in a practical way could be the process of Shared Decision Making (SDM). SDM is a form of collaboration between professionals, patients and also family members or different groups of people, in which the best available evidence is shared with the patient and contextualized when faced with the task of making health decisions (10, 14, 15, and 16).

SDM opens the possibility for the patient to learn useful information and discuss it with his or her family, health care team or physician in order to make a decision that is not only evidence-based but also appropriate to his or her social, relational and material context and uniqueness. This implies attributing to this patient a fundamental role in the construction of the diagnosis and treatment, since he/she is the one who is aware of his/her social, relational and material context. Despite the asymmetry between professional and patient, it could be said that there will be a meeting of knowledge: the professional or health team, with experience in technical/clinical knowledge and the patient and family or social circle, with experience in their subjective and social context.

This article attempts to show that these more complex versions of autonomy and vulnerability allow for a better response to people with mental illness, enriching the theory and practice of bioethics when they work together. To this end, it develops: a) the importance of the use of relational autonomy in the field of mental health; b) the dialogue between relational autonomy and vulnerability understood in layers; and c) a novel proposal of SDM that «operationalizes» relational autonomy and the metaphor of layers of vulnerability as an intermediary.

## 2. Development

### Relational autonomy and mental health

Autonomy is a fundamental concept for bioethics and research ethics (17, 18). The most widespread and widely used concept of autonomy in these fields is that of personal autonomy, and has its origins in theories or ethical paradigms whose main characteristic is self-regulation or self-determination, also understood as remaining free from interference by others. From personal autonomy, an autonomous action is defined as one performed intentionally, with knowledge, and without external control influences (19).

Personal autonomy is based on a vision of the individual that assumes him/her outside the framework of social and historical relations in which he/she is immersed (17). This conception of the individual affects the applicability of the concept in concrete situations, because the autonomy of individuals does not seem to function assuming absolute separation from others, but is exercised through and influenced by social relations and links (20, 21).

Taking into account the limitations of the concept of personal autonomy, feminist ethics presents the notion of relational autonomy (for a historical review of the concept and its authors, see Salles) (23). Although there are different conceptions of relational autonomy, all of them share a fundamental characteristic that must be considered at the moment of exercising autonomous action: interdependence (22). Interdependence maintains that social relations are a condition of possibility for autonomy

Considering a person’s autonomy from a relational perspective allows for a genuine exercise of autonomy. To the extent that there is a concrete social context (family, friends, work or educational environment), decisions are made in the company of others, considering the opinions of others or the actions of others (22). Moreover, relational autonomy makes it possible to think about which social circumstances make it possible for a person to exercise autonomous actions or which would limit them (20). And a novel feature of this concept is that it shifts the focus from protecting a person to building relationships that promote autonomy (21).

Relational autonomy does not pretend, as a theoretical and practical concept, to obstruct personal autonomy. Rather, it intends to start from it, ensure its existence and add a fundamental variable for its execution that has to do with socio-relational conditions.

In this sense, Mackenzie (24) proposes that relational autonomy can be described in three interrelated dimensions:

a) *Self-governance*, as that which concerns the internal competencies of agency, what the author calls capacity, which concerns the competence and authenticity of decisions. This would be what classically represents personal autonomy, the question of intentionality and situational awareness.

*b) Self-determination*, as the dimension in which freedoms or opportunities, or the lack of them, come into play. This is where structural and situational conditions that limit or promote autonomy, such as material, contextual and relational conditions, come into play. Self-determination is also used by those who describe personal autonomy, but in a very different way to that proposed by the author, almost in opposition, given that they understand it as the quality of remaining free of interference from other people, which they also call self-regulation.

c) *Self-authorization*, as the self-recognition of making decisions, taking responsibility for them; in other words, considering oneself as the authority to speak and act according to one’s own interests. It has a high socio-relational component, given that it is difficult to authorize or self-respect when the social context belittles or stigmatizes you. This is what the author also calls *status*; that is, to be recognized by society as a moral agent.

Therefore, for this conception of relational autonomy, there are two dimensions that are conditioned by the socio-relational variable (b and c), and one that is dependent on the individual variable (a). Personal autonomy is not only included, but is expanded by other fundamental conditions for its exercise.

The concept of relational autonomy is closely related to the exercise of mental health. If we understand mental health as the capacity of individuals to adapt to and transform reality through the resolution of the conflicts that arise in that relationship, as proposed by several authors (25, 26, 27), it is observed that the social bond, understood as what allows us to relate socially, functions as a vehicle for active adaptation to reality, which depends on the permanent reference, verification and evaluation of the outside world. This perspective is interesting, since it emphasizes that collective conditions (material, contextual and relational) are important for understanding the ways in which we maintain our health or become ill, and it also challenges us to use bioethical concepts that take into account our social constitution, the temporal variability they present and the fact that such conditions modify us.

Due to the above, it is necessary to work with a concept of autonomy that takes into account our social constitution to be used in the field of mental health. This allows, not only the possibility of considering the help of others in decision making, but also to identify which social conditions could improve the exercise of autonomy and those that are damaging it. This would function as an input to be able to work on such conditions, thus increasing the possibilities of autonomous decision making and, indirectiy, improving the person’s mental health.

Let’s look at an example. Sofia, 25 years old, was diagnosed a few months ago with an unspecified psychosis. Psychosis involves an altered perception of reality, characterized mainly by the presence of delusions and/or hallucinations. One of the main issues to be taken into account with regard to psychosis is that it generates high social disability, so that therapeutic action should be taken on this dimension, favoring socialization and sustaining ongoing social spaces.

Sofia has a good overall performance. She is in her final year of biochemistry. She practices aikido and does yoga and meditation. She also enjoys writing and painting. She lives with her mother, who is cared for by Sofia because she has severe cognitive impairment and is bedridden most of the day. Sofia has four brothers, three of whom live in the same city. She has a good relationship with the youngest of them.

The diagnosis was made after she presented a psychotic outbreak characterized by psychic and behavioral disorganization, complex auditory hallucinations and cenesthopathies, delusional interpretations of the environment, with a predominance of megalomaniacal ideas. She is admitted for the first time for treatment, which concludes after 11 days. When she was discharged, she was prescribed psychotherapy and a pharmacological scheme, which she complied with regularly.

The case is extensive (it is real), but it will be revealed through the proposed concepts. In this case, at least three social circumstances can be identified that affect in some way Sofia’s autonomy in relational terms, specifically, self-determination.

#### 1) Gender stereotypes.

The fact that she is the only one in her family taking care of her mother, who is not coincidentally the only woman, shows that gender roles, stereotyped, condition our forms of decision making. Sofia spends a lot of time taking care of her mother, a job that is not paid and that, in addition, prevents her from taking a paid job, which does not allow her to live on her own.

#### 2) Stigma associated with mental illness.

Since the diagnosis, Sofía has not been able to return to the university, since the institution claims that it was not possible for them to sustain her there, because they did not have the adequate infrastructure nor could they take care of medicated people. In turn, the older siblings have taken an attitude of infantilization and underestimation towards her, based on the lack of knowledge of what a psychosis is, since nobody ever explained to them adequately what this condition was about. Therefore, they appeal to the images they themselves have about psychosis, constructed from movies and media. This has reduced Sofia’s limited possibilities to leave her home.

#### 3) Health care model.

Sofia’s family has been omitted psychoeducation from the health care system, and the offer of therapies or interventions on her social circle has been limited.

Likewise, Sofia presents, within what we can think of as self-determination, two positive possibilities: first, the regular practice of aikido, which provides her with a concrete, well-constituted social group and a highly concentrated practice; and second, the presence of her younger brother, who has a more horizontal relationship with her sister, who understands her and is someone Sofia trusts.

On the other hand, there appear to be two conditioning factors that help to promote Sofia’s autonomy: the first is self-management, such as the fact that she engages in artistic (she writes stories and paints) and physical activities (besides aikido, she does yoga and meditation), which allow her to control several of her symptoms and sustain her subjectivity, and the second is self-authorization, since Sofia authorizes herself and intends to finish her degree and continue studying at the postgraduate level.

However, if Sofia’s situation had been observed from the perspective of personal autonomy, the focus would only have been on self-governance, disregarding the other two dimensions which, as we can see, are fundamental to think about autonomy.

## 2. The dialogue between relational autonomy and layered vulnerability

### a) Vulnerability thought in layers

Persons with mental illness or suffering have been and are considered vulnerable persons at the moment of analysing any situation in relation to inclusion in research or clinical practice (28). Vulnerability, in this context, has an important normative role in that, when it is identified, an attempt is made to provide protection or safeguards for people who could suffer harm. The problem with its use is that each of the people in such a group may experience different types of vulnerabilities, or perhaps none at all. With this sub-population approach, under the label of vulnerable, it is not possible to single out the decision-making capacities of a person or a subgroup, nor to put into context what their potential possibilities are for exercising their autonomy (29). This label, insofar as it is rigid and homogenizing, can lead to stigmatization and overprotection, which in the field of research can result in the exclusion of research subjects. Therefore, paradoxically, it ends up unprotecting them, generating, as a consequence, a scarce production of knowledge in this area (30). And in the case presented here, people suffering from mental suffering are seen as incapable of making decisions or exercising their autonomy in its entirety.

This conceptual paradigm was strongly criticized and, at times, its usefulness was dismissed because of its rigidity and because it stereotyped certain human groups (31). It was also suggested that caution should be exercised, given the essential fragility of people, because if all of them were vulnerable, then the concept would lose its usefulness, because it would no longer serve to identify subjects in need of protection (32).

However, it is necessary to recover the concept of vulnerability. There are people who find themselves in conditions that make them more susceptible to harm and who, therefore, should have special protection, safeguards and the possibility of empowerment. A proposal that refers to this recovery is the notion of layers of vulnerability (29, 30, and 32).

Vulnerability thought from the notion of layers (29, 30, and 32) makes it possible to recognize those circumstances that can generate vulnerabilities in certain people, without transforming them into permanent ones or delegitimizing the subject who suffers them. In this conceptualization, vulnerability is understood as dynamic, in close relation to the context and the specific relationships of a given moment for a given person. This approach makes it possible to identify different layers of vulnerability of a subject and to work to avoid, minimize and modify them.

This new conception explains the notion of vulnerability in terms of a dispositional property. It points out the relevance of the stimulus conditions that can «trigger» vulnerability (33). For example, a person may suffer a layer of vulnerability depending on certain circumstances; that is, the disposition is latent until a specific stimulus triggers such a layer. In other words, if there is a serious possibility that the stimulus condition will occur, it can lead to its actualization and harm the individual. Identifying the stimulus conditions is essential to neutralize, minimize or prevent such vulnerability from being expressed. Another interesting element to consider is the cascade effect that some layers of vulnerability can have. This consists of a layer that, if updated, will exacerbate existing vulnerabilities and/or generate new vulnerabilities. Identifying them is very important, since they have a great power of damage. In this sense, certain obligations also arise: not to exacerbate vulnerabilities or generate new layers of vulnerability, to eradicate them if possible and, if not, to minimize them.

In Sofia’s example, and based on the conditions that we identified as limiting her autonomy, we can think of the layers of vulnerability as a second moment, in which we specify the way in which her autonomy is being limited.

A first layer of vulnerability emerges that functions as a cascade, because, not being able to finish a university degree, it will be difficult for her to get a job, which generates economic dependence on other people. In addition, Sofia’s instances of socialization are being restricted (remember that she is in the care of her mother, and now she is being treated in an infantilizing way by her siblings, who do not allow her to engage in activities outside the home), which are very necessary for any person, but particularly necessary for Sofia. The fact of not having an external socio-affective environment limits her social performance, which again deepens her affective dependence.

Sofia, living with her mother, and now under the watchful eye of her siblings, has all the conditions she needs to live adequately, although in full economic and housing dependence, and at the cost of her unpaid work, which, in turn, wears her down, worsens her relationship with her mother and limits her recreational opportunities. But the overprotective attitude of her siblings somehow generates another layer of vulnerability, since if they discontinued her care, Sofía would be left in a situation of vulnerability in emotional, welfare and economic terms.

Under the notion of layers of vulnerability, it was pointed out that Sofia should not be thought of as belonging to a vulnerable population *per se*, but as a person susceptible to several layers of vulnerability, or to a layer of vulnerability that can be triggered and generate a cascade of vulnerabilities, and it can even be thought that if the identified layers are worked on, Sofia may no longer have vulnerabilities associated with this concept.

It is essential that these layers are identified in the context of health care, as it will contribute to the relational exercise of Sophia’s autonomy and also to an ethical medical practice. If professionals identify such layers, they can act accordingly, and accompany the psychopharmacological and psychological treatment with possible therapeutic instances. For example, collaborating in the generation and maintenance of social ties, encouraging the completion of university studies, economic emancipation, and so on. This can be taken into account and «prescribed» from the health activity; for example, inviting Sofia to day hospitals, social rehabilitation workshops and other similar instances. This could work on several levels for Sofia, to be able to leave her home and free herself from caregiving tasks; to meet other people in her situation, and for her siblings to observe that Sofia’s condition does not have the implications they assume. A dialogue could also be held with the university’s directors in an exercise of information, so that they can reinstate Sofia in her studies, given that she does not imply any risk, and to be able to give certain recommendations in case she requires any special momentary care. At the same time, a communication with the siblings can be established to enable an understanding of the meaning of their sister’s disorder, not as a permanent or total disability, but as something that can eventually be modified with the right stimulus and even that she herself can work on and improve through different activities.

These new approaches to the concept of vulnerability allow us to evaluate in a novel way how to work on, minimize or eradicate such layers of vulnerability, and leave the field open for its practical use, proposing an interesting perspective to be applied in the field of mental health.

It is important to point out that, following classical conceptions, the care approach would focus on pharmacological or psychological therapy, ignoring all the analysis that is fundamental for the prognosis of their condition and, above all, for the development of their life.

### b) Vulnerability and autonomy: the necessary dialogue

This case illustrates how the concept of vulnerability can be linked to that of relational autonomy. Relational autonomy and vulnerability, thought from the notion of layers, share fundamental points: a) both are dynamic and contextual; b) they are thought from the singularity of the subject, and c) they are not posed in a binary way (autonomous yes or no; vulnerable, yes or no). These three conceptual nodes of relational autonomy and vulnerability allow us to construct a certain conjunction of both.

Moreover, these three nodes are explained in relation. For a person to exercise autonomous actions, it is necessary to consider his or her social interdependence; that is, the relationships and links established with other people or established by external circumstances, such as co-workers, blood relatives or neighbors. In addition, social interdependence also implies other social dimensions–not only the linking/relational– that effectively condition autonomy, such as belonging to a certain socioeconomic stratum, schooling, access to information, among others. These conditioning factors can be analyzed from the notion of layers of vulnerability, in order to understand specifically if there are any layers and, if so, in what way they expose the person to distort his or her autonomy, and what actions can be taken to modify this situation. This analysis, as well as delimiting the social conditioning factors of each subject, must be carried out from the singularity of the person being analyzed; it is not possible to predetermine certain conditioning factors a priori. It must be taken into account that the conditioning factors may change from person to person, or in the same reality of the person in temporal terms. In this way, it can be seen that there is a certain gradualness and contextuality in autonomy or vulnerability, which prevents us from thinking about these concepts in a static, permanent or absolute way.

At the same time, it is possible to think of protective layers, homologous to those of vulnerability, which should not only be cared for but strengthened. In the initial development of the case, some positive qualities in terms of self-determination could be identified in Sofia. In this second moment, which happens after identifying how relationally focused autonomy is being promoted, the protective layers are concretely pointed out, which in this case could be the fact that Sofia can manage her symptoms through creative activities, which should be especially encouraged, or the fact that she has a constituted social group such as aikido. It could be added, in this instance, that it is a good time to create or propose layers of protection; for example, working with Sofía ways of socializing with social rehabilitation techniques, working in family therapy and other similar ones.

### c) Shared Decision Making (SDM): relational autonomy and the metaphor of the layers of vulnerability as an intermediary

SDM is gaining importance in health care policy, because it has been shown that, when SDM is applied, patients have more knowledge of the situation, greater confidence; therefore, they participate more, generating greater adherence and more satisfaction in general for the patient, his family, the physician or the health team, and it has even been shown that its use promotes greater efficiency in economic terms (10, 14, 16). Although the concept of SDM appeared about 40 years ago (16, 34), the discussion on whether SDM improves autonomy is still ongoing. Several authors state that SDM is a process that promotes patients’ relational autonomy, not only to value what the patient needs, but also to involve other people in the decision-making process, recovering the patient’s relational context as a co-constituent of the patient’s identity and, as such, necessarily present in the decision-making process (35, 36, and 37). In a critical position, other authors argue that the term «shared» in SDM seems to give more authority to physicians and that it does not respect the decision-making place of patients (38, 39, and 40).

For this reason several authors and researchers recommend further development around SDM, autonomy and vulnerability (10, 13, 14, 34, and 37).

SDM models are multiple, as well as their definitions. All of them have in common the presentation of SDM as a collaborative model, in which the professional presents the evidence about the patient’s health problem; the patient, for his part, presents his needs and conditions to see what treatment will be most appropriate to his problem, but also to his way of life and, then, with these two informative components (the one provided by the physician and the one provided by the patient) a joint decision is made, considering the evidence, the patient’s singularities and his socio-relational context (10, 14, 15, 16, 41).

SDM has positive effects not only on patients and physicians, but also on the health system, since it has been shown to improve its efficiency; it improves the use of services by reducing costs, while the patient adheres to the treatment —because the patient himself developed his own treatment according to his possibilities—; there is better clinical performance; there is a low rate of errors —because the decision is made jointly—; there is no «waste» of the intervention or over-consultations because the treatment could not be completed, and no complications due to non-treatment.

**Figure 1:**
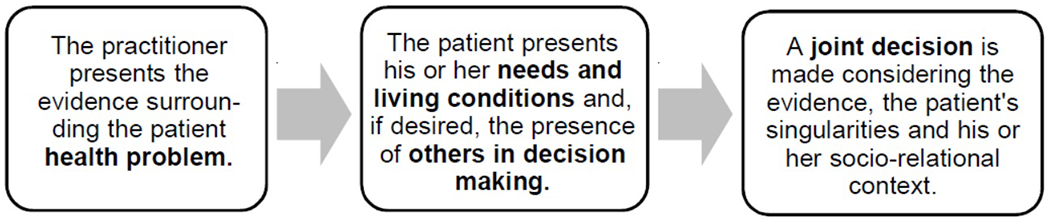
Schematic of the SDM process. Source: Own elaboration.

In schematic terms, SDM would work in this way, after defining the patient’s health problem (Figure 1):

SDM is an essential form of decision-making in the field of mental health. Decisions made in this field regarding diagnosis and treatment have a full impact on people’s way of life, since mental health as such allows us to develop our life, in its most holistic and complex form of the word. Therefore, if one suffers from a mental illness, one’s life, the way of looking at other people, at projects, at the present and the future, even one’s own outlook, among others, will be modified in the suffering, and in the way of approaching such suffering. That is why it is so important to contemplate the patient and his or her entire context, as well as his or her needs, desires and living conditions when making decisions regarding mental health.

**Figure 2:**
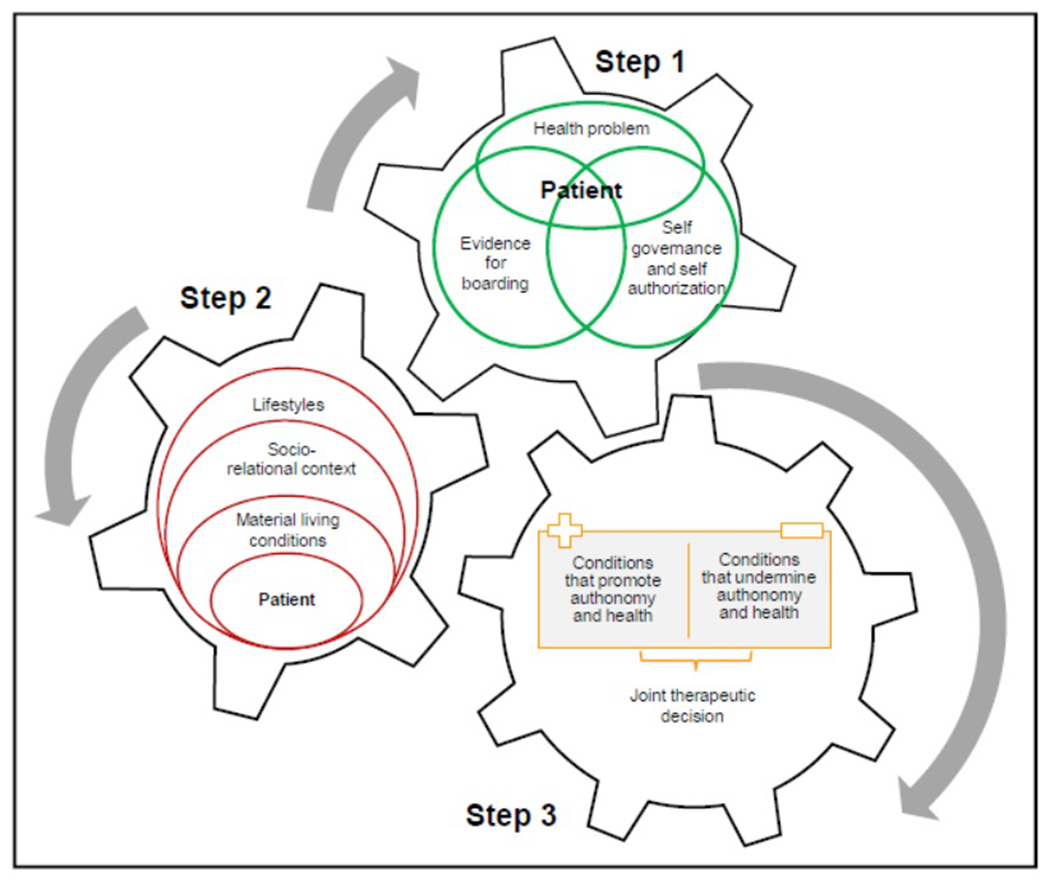
Diagram of the TDC proposal. Source: Own elaboration.

For this reason, the present proposal has to do with specifying each of the steps of the scheme in the key of relational autonomy, using vulnerability in layers as an intermediary, as conceptually worked in the previous paragraphs on these issues, particularly for the field of mental health. Figure 2 shows a SDM scheme that considers aspects that could condition autonomy in relational terms and, then, concrete situations of vulnerability. The form chosen to represent this model is not accidental: it aims to show that there is a dialectic between the steps; that when one is modified, the others are necessarily modified, at any point.

Taking Figure 1 as a reference, we will describe Figure 2.

#### Step 1

In Figure 1, the beginning has to do with the definition of a health problem by the professional and then the presentation of the evidence, diagnosis and possible treatments around that problem. Figure 2 presents similar issues, but intertwined and with the patient at the center. This can be translated into the fact that the health problem, for example, will be defined not only by the professional, but also in conjunction with the patient. It is emphasized that we are in the field of mental health, where daily life has a direct influence on it, and where intervening on daily life can improve mental health. Moreover, there are no complementary studies or any other way of gathering clinical information other than through the patient’s and/or his/her family’s account. For this reason, the patient is central in the participation of the diagnosis. At this point, the professional will be able to identify general conditions that undermine or promote autonomy. He/she will also be able to assess self-management. When starting to think about therapeutics, and starting is not a word chosen at random, because for this it is essential to complete the process, one can analyze the evidence on treatments and see what feasibility they have for the patient’s life, for example, the use of drugs (if he can handle them) and their adverse effects (if he studies and they make studying difficult, or make him sleepy); the strategies for rehabilitation, inclusion or social support; what degree of independence he has in his activities of daily living; what family or social support he has. Finally, it could be considered whether the diagnosis or some therapeutic strategy could generate some layer of vulnerability, not only to avoid it, but also to avoid exacerbating others present.

#### Step 2

In this step it is important to ask the patient to present his needs and living conditions; which suggests that this can be done by considering four dimensions that are closely related to each other: the patient’s self-authorization and then self-determination, thought of as the material conditions of life (concrete survival resources); his socio-familial relationships and his ways of life; that is, the non-voluntary groups to which he belongs. It is very important to analyze ways of life, since they involve social groups whose membership is determined not by people’s decisions to form a group (e.g., a club, voluntary association or even a social movement), but by social institutions, norms and practices, social attitudes and stereotypes, and by structural factors such as patterns of social reward and penalty, privilege and disadvantage, which shape group membership. A person belongs to a social group regardless of whether or not he or she consciously identifies with that social group. Following the feminist approach to intersectionality, a person may belong to several different social groups and therefore their actions and behavior may be institutionally restricted in different and sometimes conflictive ways (42, 43).

In a dynamic way, these issues could be explored by knowing the patient’s relationship with the social resources of his community; thinking with him how he is incorporated in his therapeutic rehabilitation strategy, based on his wishes and needs, taking into account his own strengths and weaknesses; promoting psychoeducation around the stigma associated with mental illnesses; considering moments of leisure and free time of quality and according to social desires and possibilities, without losing sight of objectives regarding pre-employment and job training.

Specifically, the following could be explored:

- Patient’s desires, needs, doubts, fears.
- Daily life of the patient.
- The characteristics of the home and maintenance.
- Functional assessment of the patient’s abilities in relation to the social resources of his community.
- Belonging to non-voluntary social groups.
- Making an ecomap or graph of the patient’s social networks.

Thus, by trying to find out about these issues, it is going to take place how the patient believes that he can adopt therapeutic alternatives; if all are valid; if, for example, you prefer another alternative because of your way of life (for example, instead of going back to university, looking for a new educational environment where there are older students, or learning a trade), all of which could behave like layers vulnerability in themselves or be the ones that contain possible layers.

#### Step 3

This is the moment when we put on the table all the layers identified by the physician and the patient; the relational elements that protect or do not protect the patient and the way in which they interact. Based on what has been identified and evaluated, we think about how to work on them in therapeutic terms, to try to avoid them or, at least, minimize them in order to guarantee the exercise of autonomy and improve the health problem. In concrete terms, we have to think about:

- Incorporate social resources that promote autonomy in the therapeutic rehabilitation strategy.
- Ensuring patient participation in the rehabilitation assessment and intervention phases.
- Ensure the participation of the family in the therapeutic rehabilitation strategy.
- Once the patient’s wishes and needs are known in step 2, motivate training in general behavioral skills, social cognition and psycho-motor skills, taking into account the patient’s own strengths and weaknesses.
- Promote psycho-education about the stigma associated with mental illness.
- To generate spaces for leisure and free time of quality, and according to social desires and possibilities.
- Establish objectives with respect to educational, pre-employment or job training.
- Consider the intervention in protective environments for the patient, so that he/she can continue with his/her activities.
- Provide follow-up and care for the patient in their usual environments if necessary.

## 4. Final considerations

The SDM process can be seen as an operationalization of relational autonomy in conjunction with vulnerability thought in layers. Each time a possible layer is identified, it is being thought of in relational or contextual terms. And this is where the richness of working both concepts together in a practical way lies. Such a SDM process is interesting, because it allows to consider the help of others in decision making and, thus, to open the game to analyze which conditions could improve the exercise of autonomy and identify those that damage it, enabling to concretely identify these conditions and work on them, to eradicate or minimize them, thus increasing the possibilities to decide autonomously and, indirectiy, to improve the mental health of the person. In this sense, it constitutes an interesting approach to recreate the clinical-assistance practices in the field of mental health and, therefore, it is very beneficial for people with mental illness, so it is necessary to promote its application.
